# Surface Functionalization of Face Masks with Cold Plasma and Its Effect in Anchoring Polyphenols Extracted from Agri-Food

**DOI:** 10.3390/molecules27238632

**Published:** 2022-12-06

**Authors:** Francesca Cicogna, Emilia Bramanti, Beatrice Campanella, Stefano Caporali, Luca Panariello, Caterina Cristallini, Randa Ishak, Niccoletta Barbani, Elisa Passaglia, Serena Coiai

**Affiliations:** 1 Institute of Chemistry of OrganoMetallic Compounds (CNR-ICCOM), National Research Council, SS Pisa, Via Moruzzi 1, 56124 Pisa, Italy; 2 Department of Industrial Engineering, DIEF, University of Florence, Via S. Marta 3, 50139 Firenze, Italy; 3 Department of Civil and Industrial Engineering, University of Pisa, Largo L. Lazzarino 1, 56122 Pisa, Italy; 4 Institute for Physical and Chemical Processes (CNR-IPCF), National Research Council, SS Pisa, Largo L. Lazzarino 1, 56122 Pisa, Italy

**Keywords:** cold plasma activation, surface functionalization, ATR-FTIR, OIT, polyphenols, eugenol, fibers morphology

## Abstract

To improve the capability of non-woven polypropylene-based fabric (NWF-PP) used for face mask production to retain active biomolecules such as polyphenols, the surface functionalization of NWF-PP–directly cut from face masks–was carried out by employing cold plasma with oxygen. The nature/structure of the functional groups, as well as the degree of functionalization, were evaluated by ATR-FTIR and XPS by varying the experimental conditions (generator power, treatment time, and oxygen flow). The effects of plasma activation on mechanical and morphological characteristics were evaluated by stress–strain measurements and SEM analysis. The ability of functionalized NWF-PP to firmly anchor polyphenols extracted from cloves was estimated by ATR-FTIR analysis, IR imaging, extractions in physiological solution, and OIT analysis (before and after extraction), as well as by SEM analysis. All the results obtained converge in showing that, although the plasma treatment causes changes–not only on the surface–with certain detriment to the mechanical performance of the NWF-PP, the incorporated functionalities are able to retain/anchor the active molecules extracted from the cloves, thus stabilizing the treated surfaces against thermo-oxidation even after prolonged extraction.

## 1. Introduction

Cold plasma treatment is a well-known and widespread technique for increasing the hydrophilicity, wettability, and adhesion characteristics of surfaces with different chemical nature and morphology. Historically, it finds application in textiles [1] where it is used to increase the dyeability of natural and synthetic fabrics, and, more recently, in the biomedical field for the immobilization of active and functional derivatives (such as cyclodextrins, dopamine, and/or antibiotic drugs) onto the uppermost layer of different substrates [2,3,4,5,6,7,8]. With reference to the functionalization of polymer-based surfaces, plasma processing is particularly versatile, safe, inexpensive, and–in the case of cold plasma–capable of significantly modifying the surface energy/features while minimizing negative impacts on the bulk properties of films or fabrics (including Non-Woven-Fabrics NWFs) that can result from high temperatures.

The final characteristics and performances of surfaces depend on the experimental conditions of the cold plasma treatment, i.e., the pressure (atmospheric or reduced), the power of the generator, the type and flow of the gas(es) chosen, and the treatment time [9,10,11]. The plasma produced by a radio frequency of 40 kHz or 13.56 MHz and direct current at low frequency of 50 kHz voltage through a pair of electrodes (glow discharge) operates at reduced pressure, while the dielectric barrier discharge (DBD, 50 Hz frequency) generally operates at atmospheric pressure. Several papers report that radiofrequency-generated plasma ensures a higher level of flexibility and uniformity of the plasma for all the gases used and better results in terms of polymer films activation as proved by surface energy and wettability measurements [1,12].

Varying some experimental parameters, different activation process steps can be observed: (1) radicals’ generation, subtly quenched by functionalizing agents in contact with the treated surface; (2) functionalization reactions producing the grafted functionalities whose nature/structure depend on the type of gas used (nitrogen, air, oxygen, ammonia, methane, and their mixtures); (3) surface indentation (due to ion bombardment), providing an increase in surface roughness.

Polypropylene nonwoven fabric (NWF-PP) find applications in agriculture, textile, water remediations, health, cosmetics, and biomedicals. NWF-PP is one of the most used materials to produce individual protection tools such as face masks and disposable gowns/coveralls that have found widespread use owing to pandemic emergency from COVID 19, which recently occurred and is still ongoing. Despite some good properties like chemical and mechanical resistance (for instance to abrasion), low cost, and the ability to be reused/recycled, NWF-PP has very weak hydrophilic and adhesion properties. Therefore, plasma modification is a suitable, easy, cheap, and low environmental cost method to overcome these shortcomings. NWF-PP has been successfully activated and functionalized with cold atmospheric or low-pressure (or vacuum) plasma in order to improve polymer–polymer interactions, wettability, printability, adhesion feature, active molecules, or dyes bonding/exhaustion [4,13,14,15,16,17,18,19,20,21,22]. Plasma treatment of PP surfaces has also been successfully used in bioconjugation to grow and produce microbial cellulose pellicles or to favor the polymerization of allylamine for the following applications in biomaterials [23,24,25]. The available literature generally relates the targeted property, which can be evaluated through contact angle measurements, peeling, surface energy measurements, and the ability to retain active and functional molecules, with the experimental parameters of the process also through statistical analysis and models [13,14,15,21,22,26,27,28,29,30,31]. Therefore, there are few or no works aimed at rating the effect of plasma treatment variables on the changes in surface chemical structure or discussing the results from detailed analytical investigations of achieved functionalities, their distribution on the surface, and their evolution over time. Some data concerning the XPS characterization [4,14,16,21,22,28,32] elucidate the possible structure of functional groups deriving from oxygen and/or nitrogen addition, roughly confirmed by ATR-FTIR spectra analysis [5,14,19,33] and with some insights in their stability [13,15,17]. SEM microscopy is generally used to highlight the surface morphological variations of fibers following the various treatments [3,20,27,29,34].

As previously proved [35], the application of polyphenolic compounds on the external surface of disposable face masks based on NWF-PP is able to increase the barrier effect of the mask itself. The results collected by using extracts from clove buds, olive leaves, and green tea confirmed the filtering effectiveness of inactivating virions; in addition, the use of plant-based polyphenols is an environmentally sustainable and friendly approach, capable to be adapted starting from local biomass waste.

The methodology adopted for the deposition of polyphenols on NWF-PP specimens, directly cut from face masks, was the direct absorption of solutions or suspensions of the active ingredients on the polymeric fabric [35]. This methodology is here compared with the advanced functionalization of NWF-PP face masks with antiviral and antioxidant polyphenols, preliminary activated with oxygen cold plasma (under vacuum) by varying operating conditions. The materials treated in the various experiments were characterized by ATR-FTIR and imaging techniques to obtain the qualitative identification and semi-quantitative determination of the functional groups generated on the NWF-PP surface by plasma treatment. The ability of NWF-PP to interact and retain bioactive polyphenol molecules–extracted from cloves–with antiviral and antioxidant capability was investigated through leaching tests in a physiological solution and by Oxidation Induction Time (OIT) test performed via DSC. NWF-PP mechanical properties were discussed with respect to morphological features after plasma treatment investigated by SEM and FTIR imaging, showing that some important topological changes on the surface of the fibers can affect their elongation and toughness, and improve the stability of anchored polyphenol.

## 2. Results and Discussion

### 2.1. Plasma Treatment of NWF-PP

#### 2.1.1. Spectroscopic Characterizations

**Figure 1:**
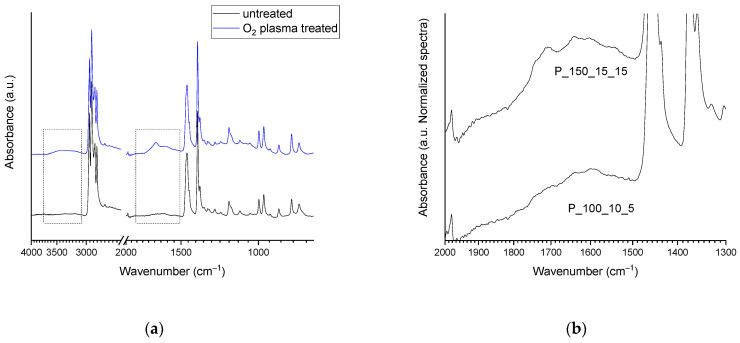
(**a**) ATR-FTIR spectra of blank (black curve) and PP_150_20_15 sample (blue curve) highlighting the region of modification (dot lines); (**b**) ATR-FTIR spectra in the region 2000–1300 cm^–1^ of sample PP_150_15_15 and sample PP_100_10_5.

ATR-FTIR analysis of all the NWF-PP samples treated with cold plasma in oxygen (Table 1) shows some characteristic signals of functionalities absent in untreated tissue (Figure 1a). In particular, -OH stretching due to alcohol and acid (as well as absorbed water) is observed between 3650 and 3100 cm^−1^, and a second broadened band–the result of the convolution of several vibrational modes–is present between 1850 and 1500 cm^−1^. The shape and the intensity of the mentioned bands depend on the experimental conditions under which the plasma treatment was carried out (Figure 1b), and their intensity increase as the power, exposure time, and oxygen flux increase.

The region between 1850 and 1500 cm^–1^ is characteristic of carboxyl and carbonyl groups (also α-β unsaturated), i.e., oxidized species resulting from oxygen grafting [36,37,38,39]. The complex signals envelope can be solved by considering the specific frequency of the C=O stretching of aldehydes and ketones, usually having characteristic absorption bands at 1725–1740 cm^–1^ and 1715 cm^–1^, respectively. The carboxylic acid -COOH absorbs at 1760 cm^–1^ and its dimer in the range of 1720–1680 cm^–1^. Therefore, absorptions at lower wavenumbers (about 1640 cm^–1^) reflect the vibrational modes of carboxyl acid dimer and -C=C double bonds (terminal vinyl) [36,39]. The oxygen-containing species are generated during the plasma treatment and by the post-treatment reactions between the active radicals created on the polymer surface by the plasma process and oxygen in the air (namely auto-oxidation).

**Figure 2:**
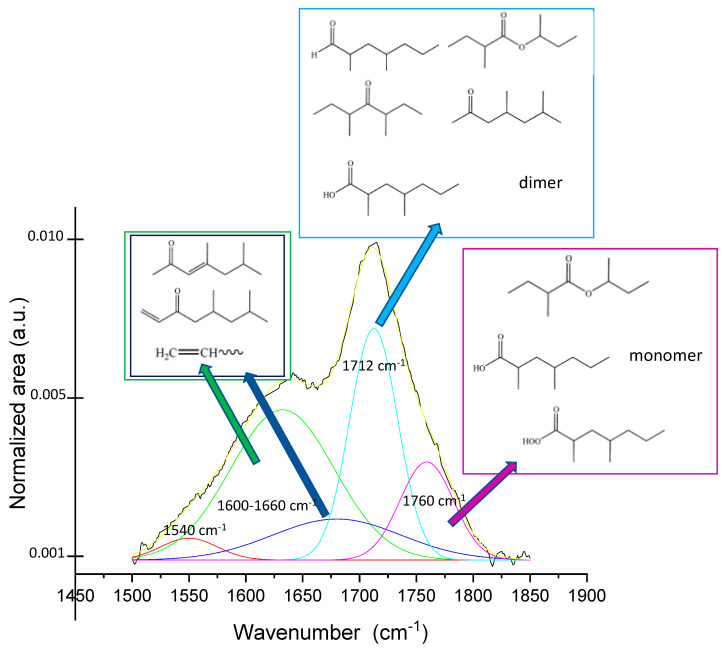
Deconvolution of signal profile in the range 1500–1900 cm^–1^ and possible attribution of each deconvoluted contribute to functionalities families of sample PP_150_15_15. Black curve is the experimental profile and yellow that obtained as best fitting.

The speciation of the different functionalities was performed by the deconvolution of the bands profile and the assignment of each component to specific functional moieties on the basis of the literature [36,37,39] (Figure 2). Five bands are observed, present with different relative intensities in all the samples. An absorption centered around 1760 cm^–1^ (the violet curve) is due to the presence of peroxyacid, carboxylic acid monomer and presumably esters (also unsaturated). A narrower band centered at 1712 cm^–1^ (the cyan curve) is attributed to the carboxylic acid dimer and the presence of aldehydes, ketones, and possibly esters.

A very broadened and generally rather intense absorption centered between 1600 and 1650 cm^–1^ (the green curve) and another wide-ranging absorption (generally weak) are attributable to the formation of unsaturated carbonyls (both aldehydes and ketones) and vinyl double bonds–the latter, probably generated by β-scission reactions of carbon radicals, possibly degrading the polymer chains on the surface. The weak contribution centered at around 1540 cm^–1^ (the red curve) is presumably due to the deformation of -NH and -NH_2_ which are generated by the reaction of reactive species (the carbon radicals) with nitrogen once the treated samples are exposed to air [38]. The survival of active species such as carbon or oxygen radicals resulted from plasma treatment (irrespective of the gas used) has been assumed and employed in post-plasma functionalization processes for the grafting of various functionalities [1,18].

**Figure 3:**
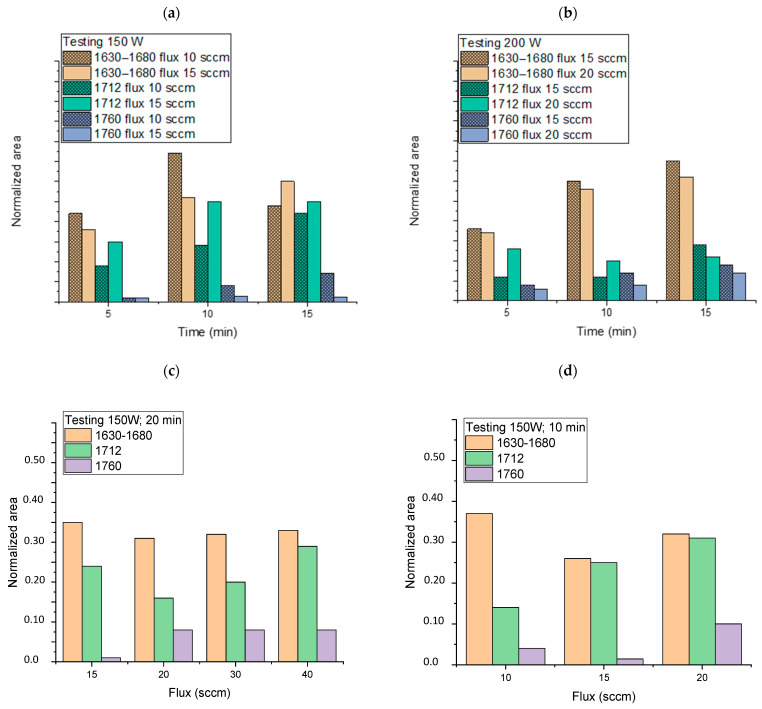
Normalized area of attributed functionalities (obtained by deconvolution as reported in Figure 2) as a function of time (**a**) at 150 W and oxygen flux of 10 and 15 sccm, and (**b**) at 200 W and oxygen flux of 10 and 15 sccm, and (**c**) as a function of oxygen flux by keeping constant the generator power (150 W) and time (20 min) and (**d**) as a function of oxygen flux by keeping constant the generator power (150 W) and time (10 min).

In order to investigate the trend in the generation of functional groups by varying the experimental parameters of the plasma treatment, the normalized ATR-FTIR spectra of all the samples (Table 1) were deconvoluted in the 1500–1850 cm^–1^ range, as described above. The areas of the main absorptions, although they cannot provide absolute quantitative information, can be correlated to the plasma operating conditions, i.e., generator power, oxygen flow, and plasma exposure time (Figure 3).

**Figure 4:**
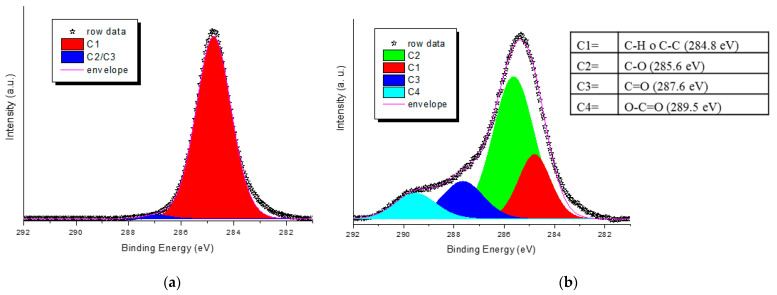
XPS spectra of the C1s core levels of NWF-PP based surface; (**a**) untreated; (**b**) sample PP_150_10_20. Inset: binding energy of deconvoluted envelope.

As a general trend, the increase in the plasma generator power does not seem to cause any change in the pattern of the intensities of the bands described moving from 150 to 200 W (Figure 3a,b). Instead, apparent lower degrees of plasma activation are observed at 200 W, especially for low exposure times and regarding the functionalities ascribed to the band at 1712 cm^–1^ (carboxylic acid dimer and saturated aldehydes and ketones). Exposure time (from 5 to 15 min), on the other hand, causes a noticeable increase in all functionalities with reference to carbonyl compounds and carboxylic acids in the dimeric form, which considerably increase their relative area. Concerning the oxygen flux, we observed that its increase has no significant effect on the 1630–1680 cm^–1^ area (owing to unsaturated functionalities) even at a high value (Figure 3c,d), but it gives a more noticeable rise of carboxylic functionalities, especially for low exposure times. The best results in terms of the content of generated carbonyl and carboxyl functionalities vs. time seem to be achieved for the following conditions: 150 W, 10 min of exposure, and flux 15 or 20 sccm. The sample PP_150_10_20, showing good absorptions in the region 1500–1850 cm^–1^ of the ATR-FTIR spectrum (normalized area were 0.32, 0.31, and 0.1 for the absorptions 1630–1680, 1712, and 1760 cm^–1^, respectively, Figure 3d) was further analyzed by XPS and compared with a blank sample to deepen the surface chemical composition. For the pristine NWF-PP, we observe a strong peak attributing to C1s (-**C**-H; -**C**-**C**-: 284.8 (eV)) and a weak peak attributing to -**C**=O functionalities (owing to contamination) (Figure 4a). The surface of the treated sample shows a completely different profile, evidencing a very strong signal due to -**C**-O (285.6 eV) with greater intensity than **C**-**C**s, indicating a significant concentration of oxygen-containing functionalities on the fiber surface (Figure 4b). This result confirms the IR analysis and the significant contribution of ketones and aldehydes to IR signals in the carbonyl region.

Several authors have demonstrated the presence of a surface layer of Low Molecular Weight Oxidized Materials (LMWOM) that are generated by combined oxidation and degradation reactions occurring during the plasma treatment [14,32,34,36]. This behavior is typical and inherent to the reactivity of PP-based materials [40,41,42] and generally begins with the formation of radicals and potentially continues even after plasma treatment, incorporating additional oxygen through exposure to air. Indeed, after three months of storage, the ATR-FTIR spectrum of several treated samples shows not only all the characteristic absorptions discussed above but also the increase in the intensity of carboxylic bands. These data confirm that the oxygen absorption and the embedding of functional groups proceed with air exposure time (Figure S1). However, when washing the sample in ethanol, most of the absorptions disappear, as depicted in the inset in Figure S1, confirming that the major part of the functional groups is due to the formation of the LMWOM layer, which is soluble in polar solvents. However, the LMWOM layer is not necessarily a boundary layer reducing the targeted adhesion or trapping effects; instead, it is reported to have a positive impact on facilitating the interfacial interactions for the desired adhesion and dyeability features, especially if the targeted functional molecules to be encompassed are able to incorporate this material [43].

#### 2.1.2. Crystal Violet (CV) Retention

**Figure 5:**
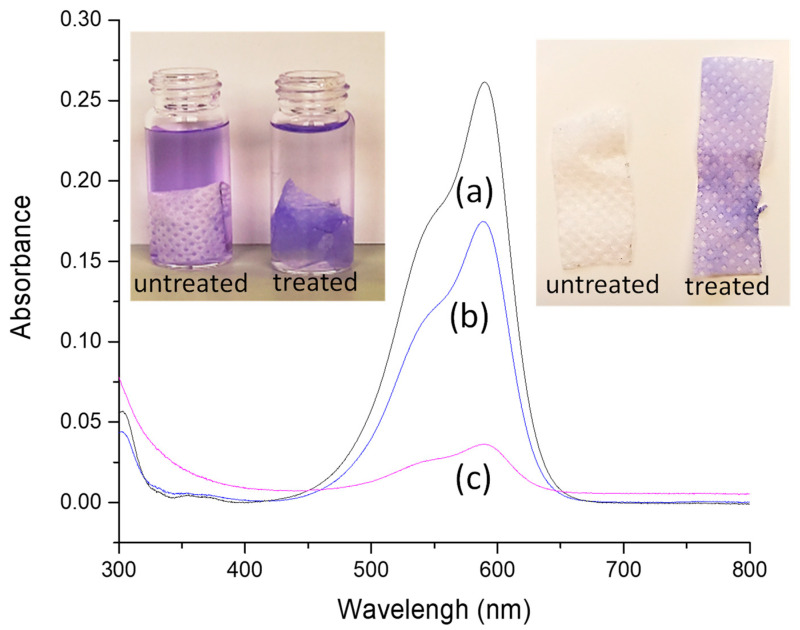
UV-vis measurements of (a) CV solution, (b) residual solution after dipped with NWF-PP, and (c) residual solution after dipped with PP_150_10_20. Inset: on the left picture of solutions with dipped samples and on the right dried samples.

In order to assess the ability of the plasma-treated sample to incorporate and/or retain a dye, the PP_150_10_20 sample (without any cleaning process) was then immersed in a buffered solution of CV. For comparison, the same solution was used for dipping the blank. From images collected during the experiment, it is evident that the plasma-treated sample can firmly anchor the dye (as demonstrated by drying the samples after dipping, see photos in Figure 5), even if the covering of the NWF-PP does not appear perfectly homogeneous with areas of colored and uncolored regular spots. Notably, while the blank absorbs only 1.2 g of CV per g of NWF, the plasma-treated sample absorbs 3.7 g of CV per g of sample, as calculated from the absorbance values of the residual solutions (Figure 5) [44]. In addition, washing with water is not able to remove the dye, whereas some leaching effect was achieved with ethanol. Therefore, although the outermost functionalized surface layer (being LMWOM) is potentially labile, the ability of the plasma-treated sample to firmly anchor the dye is evident and stated.

#### 2.1.3. Plasma-Treated NWF-PP Reactions with TEMPO Derivatives

**Figure 6:**
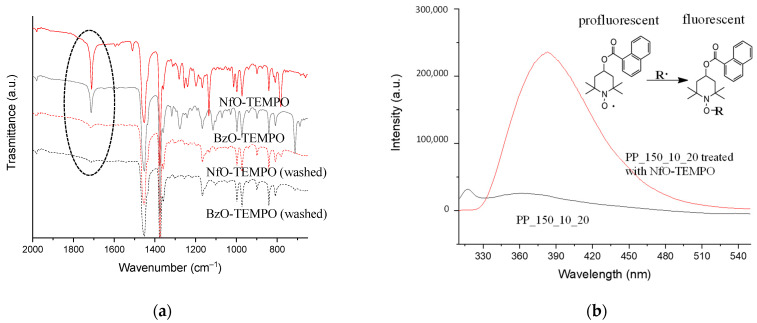
(**a**) ATR-FTIR spectra of PP_150_15_10 treated with NfO-TEMPO, BzO-TEMPO, and after washed by ethanol. (**b**) Fluorescence (emission) spectra (λ_ex_ = 290 nm) of ethanol extracts of PP_150_10_20 (pristine) and PP_150_10_20 treated with NfO-TEMPO. Inset: mechanism of reaction between NfO-TEMPO and C-centered radicals forming the alkoxyamine derivative restoring the fluorescence of naphthalene moieties.

As discussed earlier, anchoring of oxygen functionalities seems to be promoted mainly by radicals. To verify the actual ability of plasma to generate active radical species capable of re/inter-acting at a later stage (as previously demonstrated), PP_150_10_20 was immersed in BzO-TEMPO and NfO-TEMPO solutions and then washed with ethanol. BzO-TEMPO and NfO-TEMPO were used to quench C-centered radicals. Interestingly, NfO-TEMPO is diagnostic of alkoxyamine formation (the product of radical quenching) due to the fluorescence of the resulting derivative [45]. Samples were analyzed by ATR-FTIR before and after cleaning the surface with the polar solvent ethanol (Figure 6a).

Both samples treated with TEMPO derivatives show the characteristic signals of their functional groups: in particular, the band at 1709 and 1717 cm^−1^ can be associated with the carbonyl stretching of the ester group of BzO-TEMPO and NfO-TEMPO, respectively, while the bands at 781 and 710 cm^−1^ are due to the C–H out of plane bending of naphthalene and benzene [45,46,47]. Other ester absorptions are present in the spectral region between 1300 and 1130 cm^−1^, owing to C-O-C vibration modes (Figure 6a).

ATR-FTIR spectra acquired after cleaning the samples with ethanol showed a significant reduction in the signals due to nitroxide grafting and oxidation caused by the plasma treatment. The washing with the polar solvent selectively removed the surface layer of LMWOM encapsulating the carboxylic and aromatic functionalities. However, the spectra of the samples after extraction still showed weak signals due to the stretching of carbonyl groups, indicating that radical formation is not limited to the surface layers but also occurs in the bulk substrate, albeit to a lesser extent (Figure 6a). The ethanol extract of the sample functionalized with NfO-TEMPO was analyzed by fluorescence spectroscopy and compared with the ethanol extract of the plasma-treated sample (Figure 6b). Excitation at 290 nm shows an intense emission signal centered at 380 nm characteristic of the fluorescence restoring of naphthalene functionality due to the formation of the alkoxyamine bond (see the reaction scheme in the inset of Figure 6b). This result is diagnostic of alkyl radical quenching (black curve) and proves the formation of fluorescent alkoxyamine in the LMWOM layer. No comparable signal in the sample extract of a simply plasma-activated sample (red curve) was envisaged.

#### 2.1.4. Morphology and Mechanical Features

**Figure 7:**
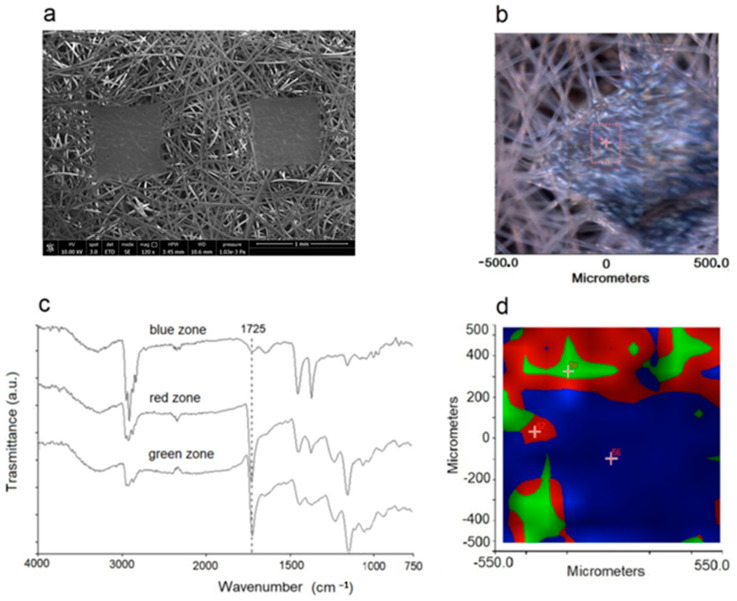
(**a**) SEM micrograph of NWF-PP. (**b**) Optical image of analyzed portion of sample PP_150_10_20. (**c**) FTIR spectra collected in the green, red, and blue zones of sample PP_150_10_20 evidenced in the (**d**) mapped imaging.

It is well known that the topology of the NWF used for face masks is not perfectly homogeneous [48]. To modulate the filtering capacity of the protection device, it is generally composed of spun laid fibers that are thermally bonded, with an ordered weave where the fused punctures–having the same size–are placed at regular distances (Figure 7a,b). The FTIR imaging analysis performed on the different zones showed that plasma activation is not able to generate homogeneous functionalization over the entire NWF surface. In particular, the analysis of the fibers showed the characteristic oxidation bands (above discussed), i.e., a broadened band centered at 1725 cm^−1^ in the spectra collected in the red and green zones of the map (Figure 7c,d). The fused spots, i.e., the blue zone of the map, show much less intense signals, meaning that the activation/oxidation process is significantly less effective. The lower local surface density and especially the greater surface area of ‘free’ fibers, those present outside the fused spot boundaries, increases plasma exposure, and facilitates oxygen uptake with a positive impact on LMWOM layer formation.

**Figure 8:**
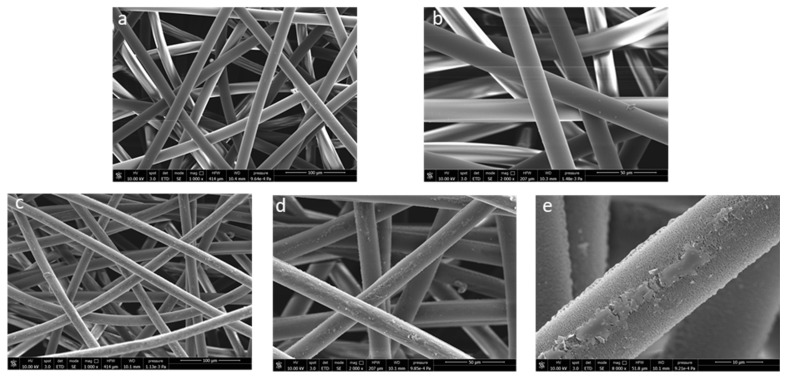
SEM micrographs at different magnifications of NWF-PP ((**a**): 1000× and (**b**): 2000×) and PP_150_10_20 ((**c**): 1000×, (**d**): 2000×, (**e**): 8000×).

The LMWOM layer was also evidenced by SEM analysis (Figure 8). Pictures reported in Figure 8 shows that the untreated fiber surface is smooth, apparently without defects (Figure 8a,b). After plasma application, a roughening was observed with the appearance of waterdrop-like or small indented crust-like swelling, together with a continuous pitting strip (Figure 8c–e). The underlying surface appears rougher and less homogenous, highlighting a significant etching phenomenon (Figure 8e) already documented [3,34]. Partial lability of the surface LMWOM layer is further demonstrated by considering a small decrease in fiber diameter (from 20.5 mm to 17.7 mm).

The plasma activation also has a significant impact on the thermal and mechanical features of NWF-PP: the treated sample shows a slight decrease in melting point and crystallinity, a remarkable decrease in onset degradation temperature, and a detriment of both stress and elongation at break (Table 2), suggesting that some bulk modification possibly occurs together with the etching phenomenon.

### 2.2. Plasma-Activated NWF-PP Surface Treatment with Cloves Extract

#### 2.2.1. Spectroscopic and Morphological Characterizations

Following our previous publication [35], untreated and plasma-treated samples were dipped in the alcoholic solution obtained from clove extraction and containing approximately 0.07g/mL^−1^ of eugenol. The degree of interaction between the polyphenol, which is polar, and the surface of the NWF-PP (apolar), can indeed be assumed to be relatively modest. The use of plasma-activated surfaces, similar to what was experienced with the CV dye, could give greater stability to the system.

The absorption-free regions of the NWF-PP ATR-FTIR spectrum of samples treated with cloves buds extract allow the clear identification of the characteristic signals of polyphenols (basically eugenol [35,49]): the -OH stretching band at 3330–3360 cm^–1^, the C=O stretching, aromatic ring deformation and aromatic C=C stretching region at 1714, 1634, 1606, 1514 cm^–1^ and the C-O-C stretching band at 1201 cm^–1^. Figure S2 shows a representative ATR-FTIR spectrum of the PP_150_10_20 sample treated with the clove bud extract. TGA experiments were used to estimate the content of extract on the surface which was 20 wt.% for untreated sample and 24 wt.% for the PP_150_10_20, determined from the % of residues as previously stated [35], (see Table 3 and Figure S3).

FTIR imaging analysis (Figure S4) revealed that the polyphenols coverage of PP_150_10_20 is not homogeneous. While the fibers appear to be functionalized by the presence of eugenol (as proved by the strong bands in the carbonyl region, shown in red on the map), their welding points show significantly less intense polyphenol diagnostic signals in the relative FTIR spectra, confirming a lower degree of coverage in accordance with the lower effectiveness of plasma activation observed above for these mapped areas.

**Figure 9:**
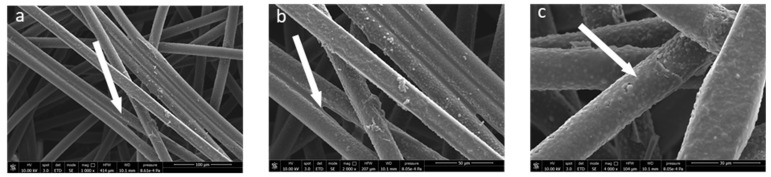
SEM micrographs at different magnifications of sample PP_150_10_20 covered by cloves extract ((**a**): 1000×, (**b**): 2000×, (**c**): 4000×).

The surface morphology of the functionalized fibers was investigated by SEM. The pictures (Figure 9) show a total fiber surface coating that is extremely homogenous, apparently without major defects and with a significant increase in the mean fiber diameter. In addition, the polyphenol layer also appears to be well adhered between fiber and fiber, acting as an adhesive/glue (see arrows in Figure 9), suggesting a very strong interaction with the fiber surface.

#### 2.2.2. Antioxidant Capability

Eugenol’s antioxidant capacity as well as its antibacterial and antiviral characteristics are well known and documented in the literature [50]. In our previous work [35], we had already observed and discussed how these characteristics can be successfully transferred to the NWF used for face masks, through simple dipping. Obtaining facial masks with such properties is of undeniable advantage in terms of extending their service life, limiting their replacement, unintentional contamination (by touching them with the hands), and facilitating their disposal. To evaluate the antioxidant capacity of the products obtained here, OIT measurements were carried out on the various samples even after prolonged extraction (five days) in a physiological solution to allow the migration/leaching of most of the polyphenol (Table 3 and Figure S5). Our previous results did not provide an accurate study of the eventual release processes (leaching) of the polyphenols over time, which, considering their polar functionalities (-OH) not interacting with non-polar surfaces of the NWF-PP, cannot be excluded.

The plasma-treated sample shows reduced stability with respect to the blank, presumably owing to the faster degradation/oxidation reaction induced by the presence of the LMWOM layer (as even proved by TGA analysis, the results of which are reported in Table 2). On the other hand, the sample covered by polyphenol does not degrade, and even after 50 min no oxidation is observed. After the extraction, we found substantial different stability of the two samples, which is significantly greater for the plasma pre-treated sample (despite the lower pristine resistance to oxidation of sample PP_150_10_20). The behavior of the material is due to a higher content of residual polyphenol, as also demonstrated by the IR spectra (Figure S6), which confirms the effectiveness of plasma treatment in anchoring the polyphenols substrates.

## 3. Materials and Methods

### 3.1. Materials

NWF-PP pieces were directly cut from face masks freely distributed from Regione Toscana during the first phase of the epidemic outbreak. Dry clove buds were purchased from a local market (produced in Italy). Deionized water was obtained from an Elga Purelab Ultra system from Veolia Labwater (High Wycombe, United Kingdom). Ethanol (HPLC grade, ≥99.8%, ≥99.8%, Sigma-Aldrich, St. Louis, MO, USA), was used as solvent for the extraction. Acetone (≥98.0%, Sigma-Aldrich) was used for dissolving 4-benzoyloxy-2,2,6,6-tetramethylpiperidine-1-oxyl (BzO-TEMPO, Fluka) and 4-(1-naphthoate)-2,2,6,6-tetramethylpiperidine-1-oxyl (NfO-TEMPO); the solutions were prepared by using 38 mg of BzO-TEMPO in 8 mL of acetone and 10 mg of NfO-TEMPO in 3 mL of acetone. Sodium acetate/acetic acid buffer (pH = 4.75) was used to solubilize hexamethyl pararosaniline chloride (crystal violet, CV); 1.2 mg of CV were used in 100 mL of buffer. Saline (or physiological solution) was prepared by dissolving 0.9 g of sodium chloride (salt), in 100 mL of deionized water.

### 3.2. Samples Preparation and Characterization

NWF-PP pieces (about 9 cm × 6 cm) were exposed to plasma treatment by using a plasma reactor (Tucano, Gambetti, Italy), which has a chamber volume of about 5.5 L, with working area 118 mm × 310 mm. The process was carried out in four steps: (i) reaction chamber from ambient pressure to 0.2 mbar; (ii) stabilization of the inner pressure (5 s); (iii) oxygen inlet and plasma reaction (during plasma treatment the total pressure ranged from 0.3 to 0.8 mbar as function of oxygen flux); and (iv) venting to ambient pressure (about 150 s). The samples were exposed to plasma treatment (both side) and characterized by ATR-FTIR. The list of the experimental conditions investigated are reported in Table 1. A selected sample (with high content of functionalities) (PP_150_10_20) was produced in larger quantity and furthermore analyzed by IR imaging, XPS, SEM, OIT, and stress–strain measurements, and the achieved results compared with those obtained for untreated NWF-PP. In addition, PP_150_10_20 was treated with 2 mL of acetone solution of BzO-TEMPO or 3 mL of NfO-TEMPO acetone solution immediately after the plasma activation, washed with fresh acetone, and dried in an oven (90 °C) for 30 min to verify the actual ability of plasma to generate active radical species capable of re/inter-acting at a later stage. Then, the sample was analyzed by ATR-FTIR. The untreated (NWF-PP) and a portion of PP_150_10_20 samples (sized about 3 cm × 4 cm) were soaked in CV buffer (for 48 h) and the resulting solutions analyzed by UV-vis spectroscopy to determine the residual concentration of CV by recording the absorbance of the solution at 590 nm.

Clove buds were pulverized with a domestic blender, and 5 gr of powder were extracted at RT with 40 mL of 3:1 (*v*/*v*) 95% ethanol:water under magnetic stirring for 12 h. Successively, the suspensions were filtered and the solution stored at −20 °C. The content of eugenol was determined by UV-Vis absorbance at 281 nm (ε = 2650 M^−1^ cm^−1^) by using a calibration curve performed with weighted amount of eugenol standard, considering that eugenol represent more than 95% of polyphenols in the aqueous extract [35].

The NWF-PP blank sample and the selected plasma-treated NWF-PP face masks (sized about 3 cm × 4 cm) were dipped in 2 mL of alcoholic clove extract and air dried before analysis by ATR-FTIR spectroscopy, IR imaging, and OIT. The samples were then extracted in a physiological solution (stirring the soaked samples for five days) and then dried in an oven; the resulting materials were analyzed by ATR-FTIR, OIT, and TGA.

### 3.3. Instrumentations

#### 3.3.1. FTIR Analysis

Infrared spectra were recorded by using a Perkin-Elmer Spectrum Two FTIR Spectrophotometer (Norwalk, CT, USA), equipped with a universal attenuated total reflectance (ATR) accessory with diamond crystal. Untreated NWF-PP and NWF-PP treated by plasma and with the extracts before and eventually after extraction (leaching tests) were analyzed in ATR mode after background acquisition. For each sample 32 scans were recorded, averaged and Fourier-transformed to produce a spectrum with a nominal resolution of 4 cm^−1^. All the ATR FT-IR spectra in the carbonyl region were deconvoluted by using four or five gaussian shaped bands having maximum at about 1550 (±20), 1630 (±20), 1680 (±20) (not always deconvoluted), 1720 (±20) and 1760 (±20) cm^−1^. The deconvolution was performed by using a NLSF method (Nonlinear Least Squares Fitter) (OriginPro 2018 software, OriginLab Corporation, Northampton MA, 01060, USA) and by optimizing for each peak the values of the area, the half width, and the maximum of the peak, starting from determined values and by varying them between a pre-determined range.

#### 3.3.2. FTIR Imaging

FTIR spectra and maps were carried out using a Perkin Elmer Spectrum Spotlight 300 FTIR Imaging System (Norwalk, CT, USA). For each sample, an area of 1 mm × 1 mm was defined to cover all the structures present in the material, and an IR image was produced using a liquid nitrogen cooled, 16-pixel mercury cadmium telluride (MCT-A) line detector at a resolution of 25 µm per pixel. One spectrum was recorded for each pixel in the µATR mode. For each sample, specific areas of interest were identified by means of the optical microscope, the ATR objective was touched on the sample, the spectra generated from the surface layers of the sample were collected, and IR spectral images were produced. The spotlight software (PerkinElmer, Norwalk, CT, USA) used for the acquisition was also employed to pre-process the spectra. Before capturing the IR image, the ZnSe window was measured as a reference, and a background spectrum was collected for each of the 16 pixels. All spectra were recorded in the mid infrared region (4000–750 cm^−1^) at 16 scans per pixel; the spectral resolution was 4 cm^−1^; the spatial resolution was 100 µm × 100 µm. The results were statistically elaborated through principal component analysis (PCA), in order to identify the zones on the map with the same spectral variability.

#### 3.3.3. UV-vis Analysis

UV-vis absorption spectrum of clove buds ethanol/water extract was recorded after suitable dilution at room temperature with JascoV750 (Cremella, Italy) and was acquired in the 700–200 nm region using 10 scans with a resolution of 1 nm.

#### 3.3.4. Fluorescence Analysis

Fluorescence emission spectra were collected by a FluoroMax4-TCSPC fluorometer (Horiba, Rome, Italy) with a xenon lamp as the excitation source: the emission spectra (λ_ex_ = 290 nm) were collected directly on ethanol extracts of PP_150_10_20 sample and PP_150_10_20 sample treated with NfO-TEMPO.

#### 3.3.5. Differential Scanning Calorimetry (DSC)

DSC measurements were performed on 5–10 mg samples under nitrogen atmosphere (nitrogen flow was 50 mL·min-1 for all the experiments) by using a Perkin-Elmer DSC-4000 (Milan, Italy) differential scanning calorimeter thermal analyzer cooled with water. Previously, the instrument was calibrated by using indium (m.p. 156.6 °C, ΔH = 28.5 Jg^−1^) and zinc (m.p. 419.5 °C). Samples were heated from 30 to 200 at 10 °C min^−1^.

#### 3.3.6. Oxidative-Induction Time (OIT) Measurements

The OIT analyses were performed by using a Differential Scanning Calorimeter DSC 4000 Perkin Elmer (Milan, Italy). The OIT measurements were carried out according to the standard testing procedure of ISO 11357-6-2008. Samples of approximately 0.5 cm × 0.5 cm were used for each measurement; the analysis cycle provides an isothermal step at 30 °C for 5 min and after a heating to 180 °C at a rate of 20 °C min^−1^ under nitrogen flow of 50 mL min^−1^. After maintaining in nitrogen for 5 min to grant the complete melting and to attain thermal equilibrium, the gas was switched to pure oxygen at a flow rate of 50 mL min^−1^. The OIT was determined by the onset of exothermic oxidation reaction.

#### 3.3.7. Thermogravimetric Analysis (TGA)

TGA of NWF-PP treated with plasma and with the polyphenols (before and after extraction) was carried out using a Seiko EXSTAR 7200 TGA/DTA instrument (Masterlab, Milan, Italy). Measurements were carried out under nitrogen flow (200 mL min^−1^) in the 30–700 °C range, at HR of 10 °C min^−1^ scanning rate, on 5–10 mg samples. The analysis of the thermograms was used to determine the temperature values corresponding to the onset and inflection point.

#### 3.3.8. X-ray Photoelectron Spectroscopy (XPS)

XPS was employed to assess the surface chemical composition of samples before and after the plasma treatment. The experiments were carried out in an ultrahigh vacuum (UHV, 10^−9^ mbar) system equipped with a VSW HAC 500 hemispherical electron-energy analyzer (model HA100, VSW Scientific Instrument, Ltd., Manchester, UK) using a non-monochromatic Mg Kα X-ray source (1.254 keV, model TA10, VSW Scientific Instrument Limited Manchester, UK) operating at 120 W power (12 kV × 10 mA). The samples were analyzed as received by fixing them to the sample holder by means of a conductive carbon tape and introduced in the UHV under inert gas (N_2_) flux and kept in the introduction chamber for at least 24 h before the measurements. Survey and high-resolution spectra were acquired in the constant analyzer energy mode (CAE) at pass energy Epas = 22 eV with a step size of 1.0 and 0.1 eV, respectively. The spectra peaks were fitted using CasaXPS software employing Gauss-Lorentz curves after subtraction of a Shirley-type background and the Binding Energy (BE) scale was calibrated taking as reference the position of aliphatic C1s component at 284.8 ± 0.1 eV.

#### 3.3.9. SEM Analysis

Scanning electron microscopy analyses were carried out at the “Centro per l’Integrazione della Strumentazione Scientifica—Università di Pisa (CISUP)” using a FEI Quanta 450 FEG-SEM (by Thermofischer Scientific, Pardubice, Czech Republic) equipped with an EDX spectrometer Bruker QUANTAX XFlash Detector 6|10, Berlin, Germany. The micrographs were recorded on the surface of NWF-PP before and after treatments. To obtain high-resolution images, all samples were Sputter coated with graphite before SEM observations.

#### 3.3.10. Stress–Strain Measurements

Tensile tests were carried out using an Instron universal testing machine model 5500R (Canton, MA, USA). The machine was equipped with a 100-N load cell and interfaced with Merlin software (INSTRON version 4.42 S/N—014733H). Rectangular specimen 4 mm × 40 mm were cut with a sharp scissor. The initial grip separation was 40 mm and the deformation rate was set to 10 mm/min.

## 4. Conclusions

NWF-PP surface, directly cut from face masks, was successfully activated/functionalized by cold plasma treatment with oxygen and successively used as a substrate to immobilize agrifood-extracted polyphenols. The study carried out under varying experimental treatment conditions reveals the formation of carbonyl and carboxyl groups and unsaturations resulting from oxygen grafting and cleavage reactions mediated by radicals occurring both during the treatment and the subsequent exposure of the plasma-treated surfaces to the air. An accurate ATR-FTIR characterization based on the deconvolution of the spectra allowed the structural identification and a comparative semiquantitative determination of grafted functionalities, evidencing some trends related to experimental parameters. The activation time seems to play a fundamental role in the degree of functionalization with reference to carbonyl compounds and carboxylic acids in the dimeric form, by increasing their content with time. The SEM and FTIR microspectroscopic topological and morphological characterization of the NWF-PP specimens showed that the functionalization is not homogeneous but reflects the peculiar structure of the NWF-PP used for the masks. The areas characterized by melting points, which are crucial to regulating the filtration capacity, are less functionalized due to the smaller surface area exposed and can consequently anchor less polyphenol. However, the presence of the active layer containing LMWOM on the surface of fibers is also clearly evidenced by SEM analysis and it has proved capable of firmly immobilizing both a dye used as a probe (CV) and polyphenols extracted from cloves. Finally, while the plasma treatment increases the content of incorporated polyphenol (which is not extractable), it weakly impacts the thermal and mechanical bulk properties of the NWF-PP to detriment of stress–strain features.

## Figures and Tables

**Table 1:** List of the experimental conditions employed for the plasma treatment of NWF-PP.

Entry	Run *	Plasma Generator Power (W)	Time (min)	Oxygen Flux (sccm)
1	PP_100_5_5	100	5	5
2	PP_100_10_5	100	10	5
3	PP_100_15_5	100	15	5
4	PP_150_5_10	150	5	10
5	PP_150_10_10	150	10	10
6	PP_150_15_10	150	15	10
7	PP_150_5_15	150	5	15
8	PP_150_10_15	150	10	15
9	PP_150_15_15	150	15	15
10	PP_150_10_20	150	10	20
11	PP_200_5_15	200	5	15
12	PP_200_10_15	200	10	15
13	PP_200_15_15	200	15	15
14	PP_200_5_20	200	5	20
15	PP_200_10_20	200	10	20
16	PP_200_15_20	200	15	20
17	PP_150_20_15	150	20	15
18	PP_150_20_20	150	20	20
19	PP_150_20_30	150	20	30
20	PP_150_20_40	150	20	40

* Samples were treated on both sides.

**Table 2:** Thermal and mechanical properties of NWF-PP and PP_150_10_20.

Run	Tm (°C) ^a^	ΔH (J g^−1^) ^a^	T_onset_ (°C) ^b^	T_infl_ (°C)	e (%) ^c^	s (MPa) ^c^
NWF-PP	161.4	100.4	424	468	65 ± 3	4.2 ± 1.5
PP_150_10_20	158.9	92.8	406	469	35 ± 3	2.7 ± 0.6

^a^ Determined based on the second heating. ^b^ Determined as the temperature with 5% of weight loss. ^c^ Measurements carried out on triplicate samples.

**Table 3:** Content of polyphenols and OIT values of samples treated with cloves extract before and after extraction in physiological solution.

Sample	% Polyphenol (from TGA) ^a^	OIT (min)
NWF-PP	0	2
PP_150_10_20	0	<1
NWF-PP_cloves	20	>50
PP_150_10_20_cloves	24	>50
NWF-PP_cloves (after extraction)	<1	4
PP_150_10_20_cloves (after extraction)	3.5	11

^a^ Determined by comparing the % of residue at 600 °C with that of extract as previously reported [35].

## Data Availability

All the data are contained within the article or Supplementary Materials.

## Supplementary Materials

The following supporting information can be downloaded at: https://www.mdpi.com/article/10.3390/molecules27238632/s1, Figure S1: Normalized FTIR-ATR spectra of blank, PP_150_10_20 sample and PP_150_10_20 sample after 3 months Inset: normalized FTIR-ATR spectra of blank and PP_150_10_20 sample after ethanol washing. Figure S2: FTIR ATR spectrum of sample PP_150_10_20 after dipping in the cloves extract; stars highlighted the main absorption bands of polyphenols. Figure S3: TGA and DTG curves of samples blank (NWF-PP) and PP_150_10_20 treated with cloves extract. Figure S4. (a) Optical image of analyzed portion of sample PP_150_10_20 treated with clove buds extract and its mapping (b); (c) FTIR spectra collected in the green, red and blue zones evidenced in the mapped imaging (b). Figure S5: OIT curves of pristine PP-NWF, PP_150_10_20, PP_150_10_20treated with cloves, PP-NWF treated with cloves and PP_150_10_20 treated with cloves after extraction. Figure S6: FTIR-ATR of samples NWF-PP_cloves and PP_150_10_20_cloves both analyzed after extraction.

## Sample Availability

Samples are available from the authors.

## Abbreviations

ATR-FTIR	Attenuated total reflectance Fourier transform infrared spectroscopy
BzO-TEMPO	4-benzoyloxy-2,2,6,6-tetramethylpiperidine-1-oxyl
CV	Crystal Violet: Hexamethyl-p-rosaniline chloride
DBD	dielectric barrier discharge
DSC	Differential scanning calorimetry
LMWOM	Low Molecular Weight Oxidized Materials
NfO-TEMPO	4-(1-naphthoate)-2,2,6,6-tetramethylpiperidine-1-oxyl
NWF-PP	Polypropylene nonwoven fabrics
OIT	Oxidation Induction Time
SEM	scanning electron microscopy
UV-Vis	Ultraviolet Visible spectroscopy
XPS	X-ray photoelectron spectroscopy
