# Supplementary material for: Surface Functionalization of Face Masks with Cold Plasma and Its Effect in Anchoring Polyphenols Extracted from Agri-Food
Source: Molecules. 2022 Dec 6;27(23):8632. doi: 10.3390/molecules27238632 (PMC9737527; doi:10.3390/molecules27238632)
Supplement: Supplementary file 1 [file molecules-27-08632-s001.zip › molecules-1999824-supplementary.pdf]

Supporting information

Surface functionalization of face masks with cold plasma and its effect in anchoring polyphenols extracted from agri-food

Francesca Cicogna[1], Emilia Bramanti[1], Beatrice Campanella[1], Stefano Caporali[2], Luca Panariello[3], Caterina Cristallini[4], Randa Ishak[3], Niccoletta Barbani[3], Elisa Passaglia[1*], Serena Coiai[1]

[1] National Research Council-Institute of Chemistry of OrganoMetallic Compounds (CNR-ICCOM), SS Pisa, Via Moruzzi 1, 56124 Pisa, Italy
[2] Department of Industrial Engineering, DIEF, University of Florence, Via S. Marta 3, 50139 Firenze, Italy
[3] Department of Civil and Industrial Engineering, University of Pisa, Largo L. Lazzarino 1, 56122, Pisa, Italy
4 National Research Council-Institute for Physical and Chemical Processes (CNR-IPCF), SS Pisa, Largo L. Lazzarino 1, 56122 Pisa, Italy
* Correspondence: elisa.passaglia@pi.iccom.cnr.it

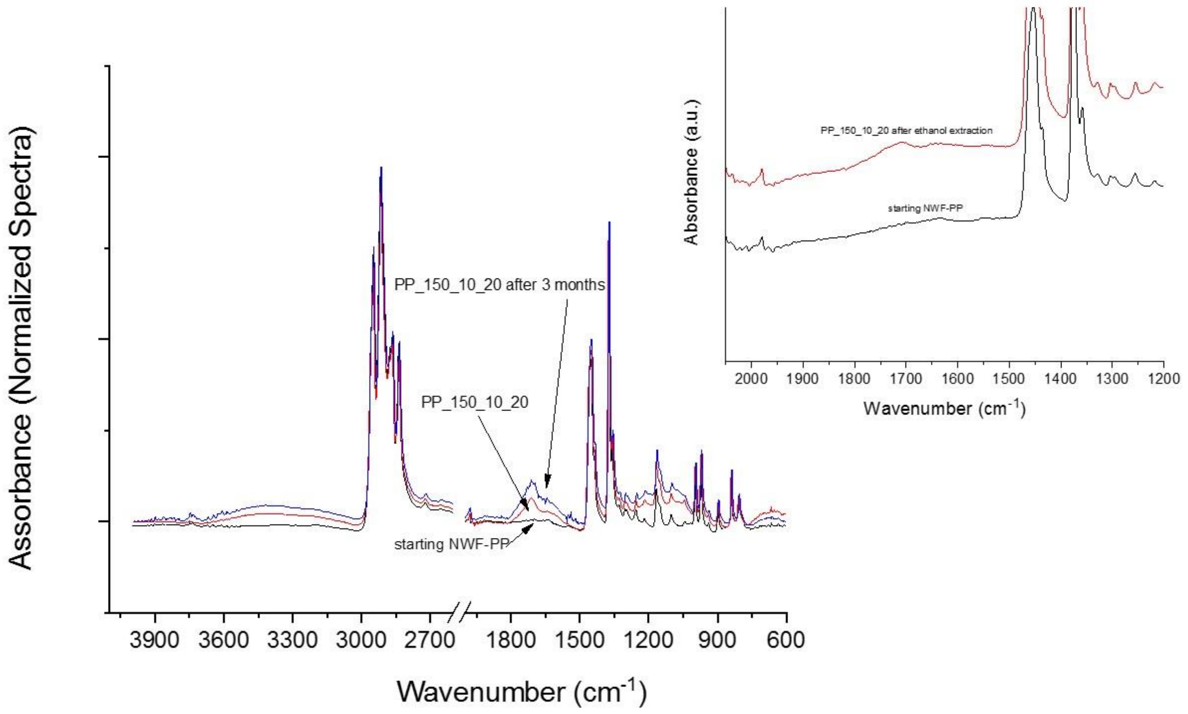

Figure S1. Normalized FTIR-ATR spectra of NWF-PP, PP_150_10_20 sample and PP_150_10_20 sample after 3 months Inset: normalized FTIR-ATR spectra of NWF-PP and PP_150_10_20 sample after ethanol washing

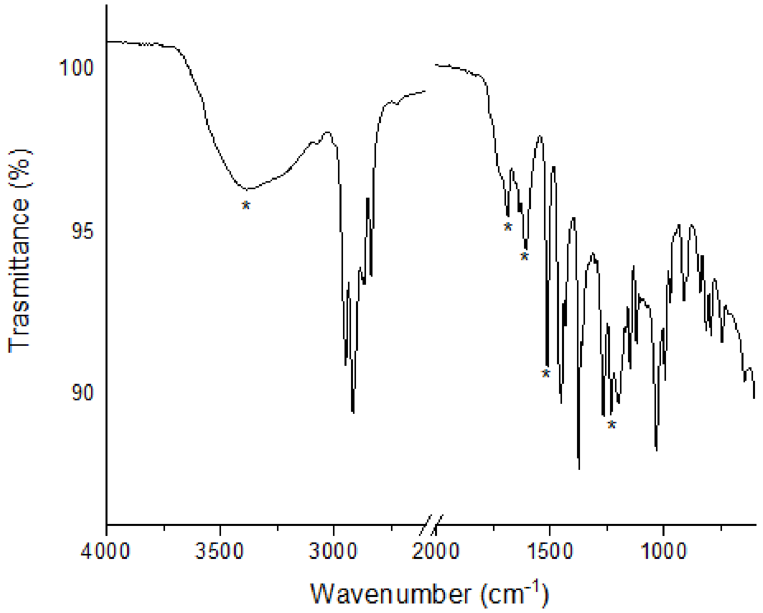

Figure S2: FTIR-ATR spectrum of sample PP_150_10_20 after dipping in the cloves extract; stars highlighted the main absorption bands of polyphenols

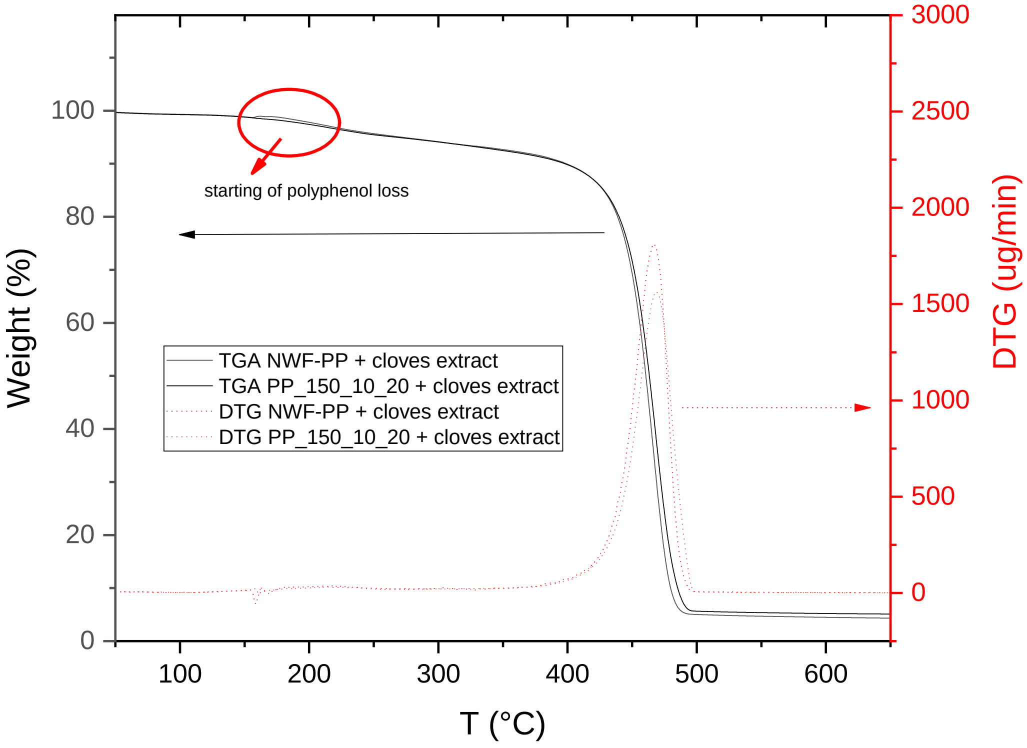

Figure S3: TGA and DTG curves of samples blank (NWF-PP) and PP_150_10_20 treated with cloves extract

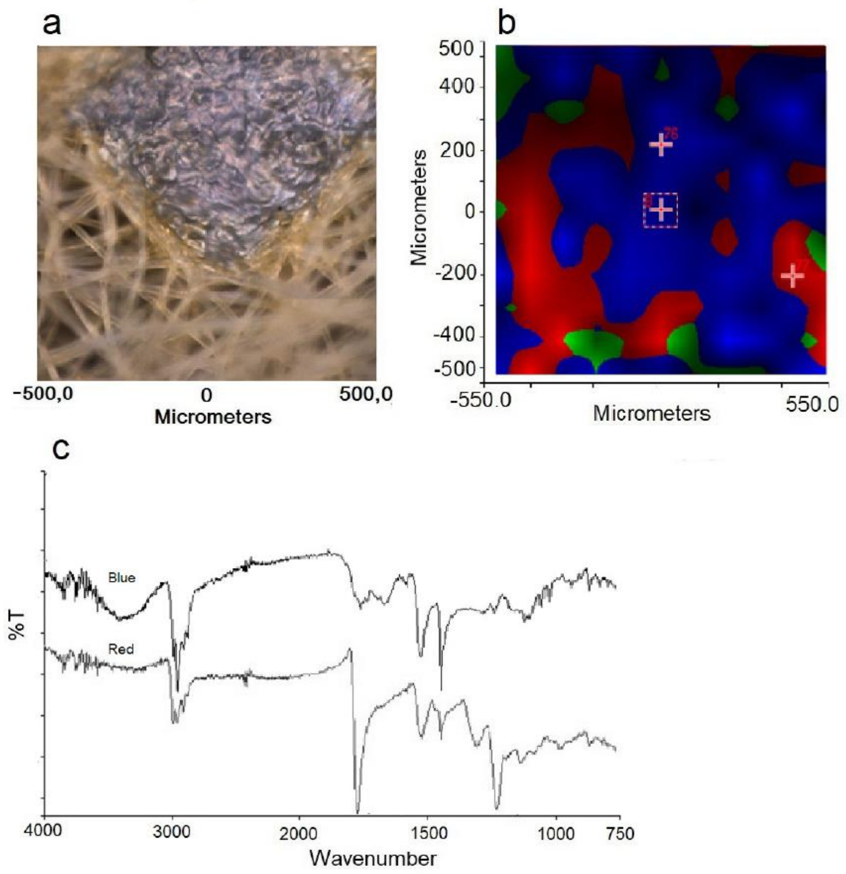

Figure S4. (a) Optical image of analysed portion of sample PP_150_10_20 treated with clove buds extract and its mapping (b); (c) FTIR spectra collected in the green, red and blue zones evidenced in the mapped imaging (b).

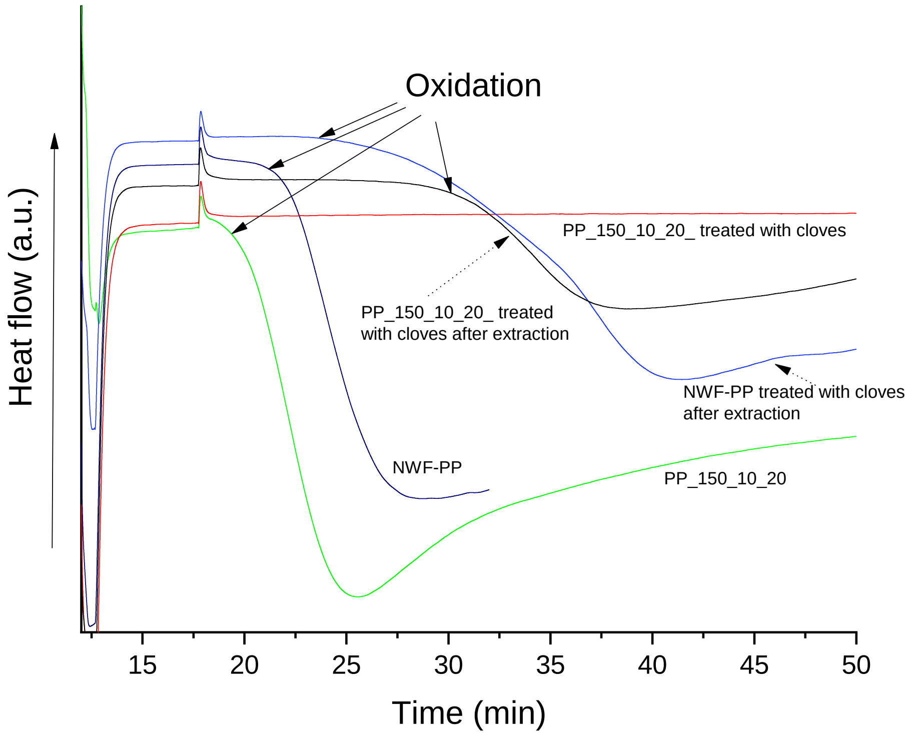

Figure S5. OIT curves of pristine NWF-PP, PP_150_10_20, PP_150_10_20_cloves, NWF-PP treated with cloves and PP_150_10_20 treated with cloves after extraction.

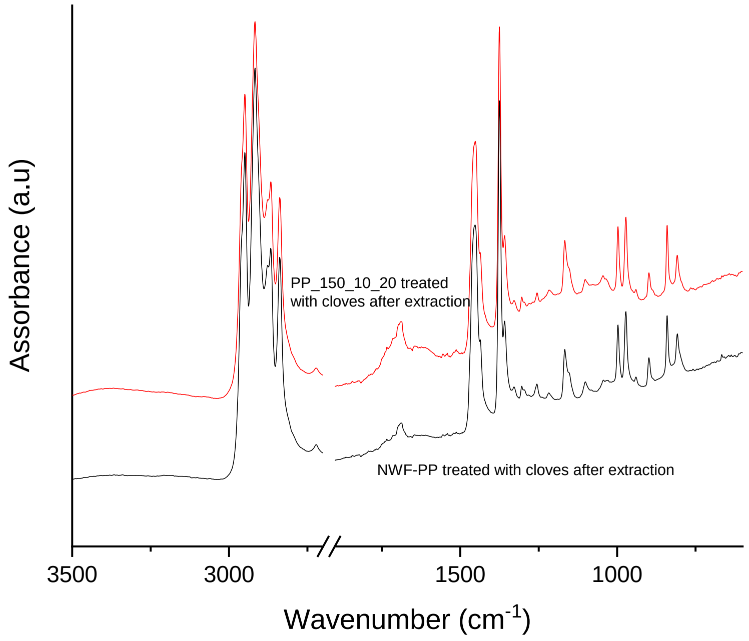

Figure S6: FTIR-ATR of samples NWF-PP_cloves and PP_150_10_20_cloves both analysed after extraction
